# Inducible clindamycin-resistant and biofilm formation in the *Staphylococcus aureus* isolated from healthcare worker’s anterior nasal carriage

**DOI:** 10.1186/s13104-024-06926-1

**Published:** 2024-09-09

**Authors:** Mahdi Dadashi Firouzjaei, Mehrdad Halaji, Sajad Yaghoubi, Peyman Hendizadeh, Maryam Salehi, Mohsen Mohammadi, Abazar Pournajaf

**Affiliations:** 1 Student Research Committee, Babol University of Medical Sciences, Babol, Iran; 2 Infectious Diseases and Tropical Medicine Research Center, Health Research Institute, Babol University of Medical Sciences, Babol, Iran; 3 Basic Sciences Department, Neyshabur University of Medical Sciences, Neyshabur, Iran; 4 Non-Communicable Pediatric Diseases Research Center, Health Research Institute, Babol University of Medical Sciences, Babol, Iran

**Keywords:** *S. Aureus*, Macrolide–lincosamide–streptogramin _B_ resistance, Biofilm, MRSA, Nasal carriage

## Abstract

**Objective:**

The purpose of this study is a new update on the resistance profile, Macrolide–Lincosamide–Streptogramin B resistance mechanisms and biofilm formation in the *Staphylococcus aureus* isolated from health care workers (HCWs) nasal carriage at a children’s teaching hospital in Babol (Northern Iran).

**Results:**

A total of 143 non-repetitive nasal swab samples were collected from volunteers, where 53.8% (n; 77/143) were HCWs, 33.6% (n; 48/143) medical students, and 12.6% (n; 18/143) resident students. The prevalence of nasal carriers of *S. aureus* was 22.4% (n; 32/143), among them, 40.6% (n; 13/32) were identified as methicillin-resistant *Staphylococcus aureus* (MRSA( carriers. Antimicrobial susceptibility testing showed that erythromycin (68.8%, n; 22/32) and ciprofloxacin (15.6%, n; 5/32) had the highest and lowest resistance rate, respectively. The frequency of resistance genes in the strains was as follows; *ermC* (n; 17/32, 53.1%), *ermA* (n; 11/32, 34.4%), *ermB* (n; 6/32, 18.7%), *ereA* (n; 3/32, 9.4%). Moreover, 50.0% (n; 16/32), 28.1% (n; 9/32) and 21.8% (n; 7/32) of isolates were strongly, weakly and moderately biofilm producer, respectively. Macrolides-lincosamides-streptogramins B (MLSB) antibiotic resistance among *S. aureus* isolates from HCWs nasal carriage have found significant prevalence rates throughout the globe. It is crucial to remember that the development of biofilms and MLS B antibiotic resistance are both dynamic processes.

## Introduction

*Staphylococcus aureus*, a catalase- and coagulase Gram-positive cocci is a prominent pathogenic microorganism that can lead to a multiple infections from minor skin and soft tissue infections (SSTIs) to severe and potentially fatal diseases [1]. The nostril is the most common carriage site for *S. aureus* and anterior nasal carriers are at high risk of developing *S. aureus* infections. Human colonization with *S. aureus* occurs in the first days of life [2]. Nasal carriers are divided into transient and permanent. Antibiotic resistance and biofilm formation are important factors in maintaining the carrier state [3].

Macrolide, lincosamide and streptogramin B (MLS_B_) are effective as a limited and alternative treatment regimen in Staphylococcal infections, especially in SSTIs. Multiple mechanisms have been identified that confer resistance to MLS_B_ antibiotics. These mechanisms include the presence of an active efflux pump encoded by the *msr* gene, drug inactivation by the *lun* gene, and the presence of the *erm* cluster, which induces changes in the ribosomal binding site via methylation and/or point mutation [4–7]. The *msrA* gene has been found to be present in *S. aureus* and is responsible for the ATP-dependent transport of erythromycin and streptogramin B out of the cell [6]. Also, *msr* plasmid genes encoding macrolide efflux pump have been described in these bacteria [6].

Biofilm, an extracellular polysaccharide matrix that surrounds bacteria, is one of the important survival and resistance factors in the carriage. The presence of polysaccharide intercellular adhesive (PIA) encoded and regulated by the intercellular adhesion operon (*ica* ADCB) is essential in biofilm formation [8]. The operon consists of three components: a N-acetylglucosamine transferase (*icaA* and *icaB*), a predicted exporter (*icaC*), and a deacetylase (icaD).

Understanding the mechanisms of antimicrobial resistance and biofilm formation in *S. aureus* nasal carriers can offer valuable insights for enhancing infection control measures and improving clinical treatment strategies in the future [9]. Therefore, the purpose of this study is to provide a new update on the resistance profile, MLSB resistance mechanisms, and biofilm formation in *S. aureus* isolated from health care workers (HCWs) nasal carriage at a children’s teaching hospital in Babol, Northern Iran.

## Main text

### Materials and methods

#### Study design, sampling and laboratory identification

The cross-sectional study was performed with the committee ethical number of IR. MUBABOL.HRI.REC.1400.159 from the one-year period of time (2022) at the Amirkola children’s teaching hospital (Babol, north of Iran). Exclusion criteria was HCWs who received antibiotics for the previous two weeks or those suffering signs and symptoms of upper respiratory tract infections.

#### Collection and processing of nasal swabs

A single nasal sample was obtained from each participant, by gently inserting a swab into their nostril and rotating it three times. The swabs were then transported to the laboratory under sterile conditions. Following this, the samples underwent culturing on Mannitol agar that had been supplemented with 7.5% sodium chloride (Merck Co., Germany), and were then incubated for a period of 24 h at a temperature of 37˚C. Standard microbiological and biochemical methods were employed to identify all resulting colonies. PCR of *nuc* gene (encoding thermonuclease) was used to confirm *S. aureus* strains [4, 10].

#### Antimicrobial Susceptibility Testing (AST)

In the current study, the Kirby-Bauer method was utilized in adherence to the guidelines set forth by the Clinical and Laboratory Standards Institute (CLSI document M100, 28th ed), for conducting antibiotic susceptibility testing (AST). For this, Mueller-Hinton agar plates (Merck, Darmstadt, Germany) were used, and the disk agar diffusion technique was employed to test the following antibiotics: ampicillin (AMP; 20 µg), erythromycin (ERY; 15 µg), gentamicin (GM; 10 µg), clindamycin (CD; 2 µg), ciprofloxacin (CIP; 5 µg), mupirocin (MUP; 5 µg), trimethoprim-sulfamethoxazole (SXT; 5 µg), tetracycline (TET; 30 µg), and cefoxitin (FOX; 30 µg) (Padtan-Teb, Iran). The methicillin-resistant *Staphylococcus aureus* (MRSA) isolates were screened based on resistance to cefoxitin (30 µg) discs (MAST, UK) by the disc diffusion method according to the CLSI guidelines *S. aureus* ATCC 25,923 was used as a quality control.

#### Determination of inducible resistant phenotypes

To identify resistant phenotypes, a double disk test was conducted by placing ERY and CD disks 20 mm apart as previously described [11].

#### Crystal violet biofilm formation assay

Biofilm production ability was assessed using 96-well flat bottom microtiter plate procedure as previously described.

#### Molecular detection of resistance determinants

Bacterial cells were lysed as follows: five pure colonies liquefied in a 25 µl of 0.25% sodium dodecyl sulfate (SDS)–0.05 N NaOH solutions and heated for 15 min. After adding 200 µL of ddH2O to the microtube, 5 µL of the diluted mixture was used in the PCR method. Successful DNA isolation was verified via agarose gel electrophoresis. Multiplex-PCR assay was performed by DNA amplification device (Eppendorf, Germany) to detect the *icaA*, *icaB. icaD*,* ermA*,* ermC* ,*ereA*,* msrA*,* msrB* using the specific primers (Table 1) [4, 12].

**Table 1:** The primer sequences used in this study

Target Genes	Primer sequences (5’→3’)	Tm (ºC)	Length	Product size (bp)	Ref
*nuc*	F;5ʹ -GCGATTGATGGTGATACGGTT-3ʹ	58.8	21	279	[10, 11]
	F;5ʹ -AGCCAAGCCTTGACGAACTAAAGC-3ʹ	63.9	24		
*mecA*	F;5ʹ - TCCAGATTACAACTTCACCAGG-3ʹ	57.7	22	162	[4, 11]
	R;5ʹ -CCACTTCATATCTTGTAACG-3ʹ	51.4	20		
*ermA*	F;5ʹ -TATCTTATCGTTGAGAAGGGATT-3ʹ	54.7	23	139	
	R;5ʹ -CTACACTTGGCTTAGGATGAAA-3ʹ	55.8	22		
*ermB*	F;5ʹ -CCGTTTACGAAATTGGAACAGGTAA-3ʹ	60	25	360	
	R;5ʹ -GAATCGAGACTTGAGTGTGC-3ʹ	56.5	20		
*ermC*	F;5ʹ -ATCTTTGAAATCGGCTCAGG-3ʹ	55.8	20	295	
	R;5ʹ -CAAACCCGTATTCCACGATT-3ʹ	56.1	20		
*ereA*	F;5ʹ -AACACCCTGAACCCAAGGGACG-3ʹ	64.7	22	426	
	R;5ʹ -CTTCACATCCGGATTCGCTCGA-3ʹ	62.7	22		
*ereB*	F;5ʹ -AGAAATGGAGGTTCATACTTACCA-3ʹ	57.3	24	546	
	R;5ʹ -CATATAATCATCACCAATGGCA-3ʹ	54.3	22		
*msrA*	F;5ʹ -TCCAATCATTGCACAAAATC-3ʹ	52.7	20	163	
	R;5ʹ -AATTCCCTCTATTTGGTGGT-3ʹ	53.8	20		
*msrB*	F;5ʹ - TATGATATCCATAATAATTATCCA-3ʹ	48.4	24	595	
	R;5ʹ - AAGTTATATCATGAATAGATTGTCC-3ʹ	52.8	25		
*icaA*	F;5ʹ -ACACTTGCTGGCGCAGTCAA-3ʹ	63.2	20	188	[13]
	R;5ʹ -TCTGGAACCAACATCCAACA-3ʹ	57	20		
*icaB*	F;5ʹ -AGAATCGTGAAGTATAGAAAATT-3ʹ	51.7	26	880	
	F;5ʹ -TCTAATCTTTTTCATGGAATCCGT-3ʹ	56.4	24		
*icaC*	F;5ʹ -ATGGGACGGATTCCATGAAAAAGA-3ʹ	60.6	24	1066	
	F;5ʹ -TAATAAGCATTAATGTTCAATT-3ʹ	47.8	22		
*icaD*	F: 5’- ATGGTCAAGCCCAGACAGAG − 3ʹ	59.4	20	198	
	R: 5’- AGTATTTTCAATGTTTAAAGCAAATAC-3ʹ	54.4	27		

PCRs were conducted in an Eppendorf Co. (Germany) master cycler gradient, with a final reaction volume of 25 µl composed of 2.5 µl of template DNA, 13.5 µl of Taq DNA Polymerase Master Mix RED (Ampliqon, Stenhuggervej, Odense M, Denmark), 1.0 µl of each primer, and 7.0 µl of ddH_2_O water.

### Statistical analysis

Statistical analysis in this study was carried out with SPSS software version 22.0 (IBM, Armonk, NY, USA), and the chi-square test was utilized to compare the data related to biofilm formation and resistance genes. A significance level of less than 0.05 was considered statistically significant.

## Results

A total of 143 non-repetitive nasal swab samples were collected from volunteers, where 53.8% (n; 77/143) were HCWs, 33.6% (n; 48/143) medical students, and 12.6% (n; 18/143) resident students.

### Antimicrobial Susceptibility Testing (AST)

In general, the prevalence of nasal carriers of *S. aureus* was 22.4% (n; 32/143), among them, 40.6% (n; 13/32) were identified as MRSA carriers (Table 2). AST showed that the highest and lowest resistance rate were related to ERY (68.8%, n; 22/32) and CIP (15.6%, n; 5/32), respectively. All FOX-resistant strains carried the *mecA* gene and were considered as MRSA.

**Table 2:** The possible risk factors associated with the nasal carriage of MSSA and MRSA among the study participants

Characteristics	Variables	Number (%)	MSSA (n; 19)		MRSA (n; 13)	
			Positive *n* (%)	*P*-value	Positive *n* (%)	*P*-value
Gender	Male	88 (61.5)	11 (57.9)	0.256	9 (69.2)	0.070
	Female	55 (38.5)	8 (42.1)		4 (30.7)	
Age (Years)	22–32	38 (26.6)	5 (26.3)	0.192	3 (23.1)	0.061
	33–43	59 (41.3)	9 (47.4)		6 (46.2)	
	44–58	46 (32.2)	5 (26.3)		4 (30.7)	
Antibiotic use	Yes	32 (22.4)	7 (36.8)	0.032	4 (30.7)	0.051
	No	111 (77.6)	12 (63.2)		8 (61.5)	
Hospitalization	Yes	16 (11.2)	2 (10.5)	0.021	1 (7.7)	0.011
	No	127 (88.8)	17 (89.5)		12 (92.3)	
Occupation	Nursing	52 (36.4)	8 (42.1)	0.041	5 (38.5)	0.012
	Laboratory	9 (6.3)	3 (15.7)		2 (15.4)	
	Service force	3 (2.1)	0 (0.0)		0 (0.0)	
	Kitchen staff	5 (3.5)	0 (0.0)		0 (0.0)	
	Security	8 (5.6)	2 (10.5)		1 (7.7)	
	Residents	18 (12.6)	2 (10.5)		2 (15.4)	
	Medical students	48 (33.6)	4 (21.1)		3 (23.1)	
Patient contact	Yes	120 (83.9)	14 (73.7)	0.036	10 (76.9)	0.043
	No	23 (16.1)	5 (26.3)		3 (23.1)	
Comorbidities	DM	8 (5.6)	6 (26.3)	0.051	3 (23.1)	0.064
	HL	3 (2.1)	2 (10.5)		3 (23.1)	
	Thyroid disorders	10 (6.9)	8 (42.1)		4 (30.7)	
	Heart failures	5 (3.5)	3 (15.7)		3 (23.1)	
Any history of infection with COVID-19	Yes	106 (74.1)	12 (63.2)	0.027	8 (61.5)	0.036
	No	37 (25.8)	7 (36.8)		5 (38.5)	

### Results of biofilm formation

According to our results, 50.0% (n; 16/32), 28.1% (n; 9/32) and 21.8% (n; 7/32) of isolates were strongly, weakly and moderately biofilm producer, respectively. The MRSA isolates exhibited significantly higher biofilm production compared to Meticillin-Sensitive *Staphylococcus aureus* (MSSA).

### Results of resistance determinants

As you can see in Table 3, the diversity of resistance genes in the strains was as follows; *ermC* (n; 17/32, 53.1%), *ermA* (n; 11/32, 34.4%), *ermB* (n; 6/32, 18.7%), *ereA* (n; 3/32, 9.4%). The genes of *ereB*, *msrA*, and *msrB* were not found in any isolate. Also, *ereA* gene was present only in MRSA strains. On the other hand, the prevalence of biofilm coding genes were as follows; *icaD* (n; 26/32, 81.3%), *icaA* (n; 22/32, 68.7%), *icaC* (n; 19/32, 59.4%) and *icaB* (n; 14/32, 43.7%). All MRSA strains carried *icaA* gene.

**Table 3:** Biofilm formation, resistance pattern and gene profile in the collected isolates

Strains	Collected resource	AST results	Gene profiling	Biofilm formation	Inducible resistance types
1	Nursing	ERY, AMP, CD, MUP, FOX, TET, SXT, CIP	*nuc/mecA/icaA/ icaD/ icaC/ ermC/ ermA*	Strong	cMLSB
2	Residents	ERY, CD, MUP, FOX, TET, GM, SXT, CIP	*nuc/mecA/icaA/ icaD/ icaC/ ermC/ ermB*	Strong	ND
3	Medical students	AMP, CD, FOX, TET, GM	*nuc/mecA/icaA/ icaD/ icaC/ ermC/ ermA*	Strong	ND
4	Nursing	ERY, MUP, TET	*nuc/ icaA/ icaC/ icaB/ ermC*	Moderate	iMLSB
5	Residents	ERY, MUP, FOX, CIP	*nuc/mecA/icaA/ icaD*	Moderate	ND
6	Nursing	ERY, AMP, FOX, GM, CIP	*nuc/mecA/icaA/ icaD/ icaC/ icaB/ ermC/ ermA/ ermB*	Strong	ND
7	Medical students	ERY, AMP, CD	*nuc/ icaA/ icaD/ ermA*	Moderate	ND
8	Medical students	ERY, MUP, GM	*nuc/ icaC/ icaB/ ermA/ ermB*	Strong	ND
9	Nursing	AMP, CD, FOX, CIP	*nuc/mecA/ icaA/ ermC*	Weak	ND
10	Laboratory	ERY, MUP	*nuc/ icaA/ icaC/ ermC*	Weak	ND
11	Nursing	AMP, CD, FOX, TET	*nuc/mecA/icaA/ icaD/ icaC/ icaB/ ermC*	Strong	ND
12	Residents	ERY, AMP, CD	*nuc/ icaA /icaD/ icaC/ ermB*	Strong	iMLSB
13	Nursing	AMP, MUP	*nuc/ icaD/ icaB*	Weak	ND
14	Nursing	ERY, CD, MUP, FOX, TET	*nuc/mecA/ icaA/ icaC/ ermC/ ereA*	Strong	ND
15	Laboratory	AMP, MUP	*nuc/ icaA /icaC/ ermC/ ermA*	Strong	ND
16	Laboratory	ERY, AMP	*nuc/ icaA/ icaD/ icaC/ icaB*	Moderate	ND
17	Medical students	ERY, CD, TET	*nuc/ icaD/ icaB*	weak	ND
18	Security	ERY, AMP, TET	*nuc/ icaA/ icaC*	weak	ND
19	Nursing	AMP, MUP, TET	*nuc/ icaA /icaD/ ermA/ ermB*	Strong	ND
20	Laboratory	ERY, AMP, CD, FOX, TET	*nuc/mecA/icaA/ icaD*	Weak	ND
21	Residents	ERY, AMP, CD, FOX	*nuc/mecA/icaA/ icaD/ icaB*	Strong	ND
22	Nursing	ERY, AMP, TET	*nuc/ icaC/ icaB/ ermC*	Weak	iMLSB
23	Laboratory	AMP, CD, MUP, FOX, SXT	*nuc/mecA/icaA/ icaD/ ereA*	Strong	ND
24	Security	ERY, CD, MUP, FOX, SXT	*nuc/mecA/icaA/ icaD/ icaC*	Strong	ND
25	Medical students	AMP, CD	*nuc/ icaD/ icaC/ ermC*	Moderate	MS
26	Nursing	CD, MUP	*nuc/ icaC/ icaB*	Weak	MS
27	Nursing	ERY, AMP, GM	*nuc/ icaA/ icaD/ ermA*	Moderate	iMLSB
28	Medical students	ERY, MUP	*nuc/ icaB/ ermC/ ermA*	Moderate	iMLSB
29	Nursing	CD, MUP, SXT	*nuc/ icaD/ ermC*	Weak	ND
30	Nursing	ERY, AMP, CD, FOX, GM	*nuc/mecA/icaA/ icaD/ icaC/ icaB* /*ermC/ ermA/ ereA*	Strong	cMLSB
31	Medical students	ERY, AMP, GM	*nuc/ icaD/ icaB/ ermC/ ermB*	Strong	iMLSB
32	Security	ERY, CD, MUP, SXT	*nuc/ icaD/ icaC/ icaB/ ermC*	Strong	cMLSB

Erythromycin (ERY), clindamycin (CD), gentamicin (GM), ciprofloxacin (CIP), tetracycline (TET), ampicillin (AMP), mupirocin (MUP), cefoxitin (FOX), and trimethoprim-sulfamethoxazole (SXT) and ND; not defined

The data indicated that 34.4% (*n* = 11/32) of the isolates demonstrated resistance to both CD and ERY. Specifically, 9.4% (*n* = 3/32) of the strains displayed a resistant phenotype to cMLSB (i.e., resistant to both ERY and CD), 18.8% (*n* = 6/32) showed inducible resistance iMLSB (i.e., resistant to ERY but susceptible to CD), and 6.3% (*n* = 2/32) of the isolates had the MS phenotype (i.e., susceptible to ERY and resistant to CD).

## Discussion

In fact, about 20–30% of humans can carry this organism continuously and asymptomatically. Therefore, nasal carriers can increase the risk of infection transmission, which leads to the serious infections, especially in hospitalized patients and immunocompromised, which is linked to increased risk of death and prolonged hospital stays [13–15]. In the present study, the prevalence of *S. aureus* nasal carriers was 22.4% (n; 32/143), of which 40.6% (n; 13/32) were MRSA. Sedaghat et al., (2017) showed that the MRSA rate in 272 collected nasal swabs was 13% [16]. Danelli et al.. (2020) found that 42.9% of 324 nasal samples were determined to be *S. aureus*, with 28.8% of those being MRSA [17]. Fard-Mousavi et al., (2015) showed that of 813 subjects screened, 10.2% (n; 83), 10.6% (n; 86) and 79.2% (n; 644) were persistent, transient and non-carriers, respectively [18]. These differences can be the result of the study population, the place of sampling (specialized hospital compared to general hospitals) and people’s awareness of personal medical-hygiene. The rate of colonization was significantly higher in people who did not use antibiotics at least in the last 3 months (0.03 and 0.05 for MSSA and MRSA, respectively). Significantly, colonization was more in people who were in contact with the patient, which suggests an increase in the incidence of iatrogenic disease. The highest colonization rates of MSSA and MRSA were respectively in nurses (42.1% and 38.5%) and then medical students (21.1% and 23.1%). In a contrast study at the Brazil, Danelli et al.. (2020) demonstrated that males and students had a significantly higher prevalence of *S. aureus* carriage (OR = 2.898); However, no factors were found to be correlated with the carriage of MRSA [17].

A high prevalence (40.0%) of MRSA from nasal-carrier HCWs was reported from Ghana in 2020 [19]. From a reported of Moghadam et al., (2015) out of 270 nasal swabs collected from HCWS, 14.4% of *S. aureus* were detected [20]. Remarkably, there was a notable discrepancy observed in terms of MRSA carriage in relation to gender (*P* = 0.041) and occupation (*P* = 0.034). Pourramezan et al., (2019) showed that the incidence of *S. aureus* and MRSA in the nasal cavities of HCWs were 39.8% (n; 53/133) and 22.5% (n; 30/ 133), respectively [21].

However, the observed differences in *S. aureus* and MRSA carriage rate in the country and other parts of the world can be attributed to variations in sample size, identification methods and local infection control polices [22, 23].

On the other hand, several probable factors contribute to the high prevalence of MRSA among HCWs. These include inadequate cleaning and disinfection protocols, high patient-to-staff ratios that may result in lapses in hygiene practices, and the frequent interaction of HCWs with patients who are positive for MRSA as opposed to those who are negative for MRSA, particularly in intensive care units or during medical procedures [24, 25].

As a highlight achievement, the resistance to MUP -a topical anti-staphylococcal ointment used for eradication of nasal carriage was 50.0%. Tabandeh et al., (2022) and Moghadam et al., (2015) showed that 14.4% (n; 14/97) and 29.4% (n; 5/17) isolates were resistance to MUP [20, 26]. On the other hand, in the study of Sedaghat et al., (2018) and Askarian et al., (2009) no isolates were resistant to MUP [16, 27]. This inconsistency in the results can be a related to the rational prescription of antibiotics, geographical distance, place and year of the study. The presence of cMLSB, iMLSB, and MS phenotypes were detected in 9.4%, 18.8%, and 6.3% of the isolates, respectively, as revealed by the D-test. Also, 53.1%, 34.4%, 18.7% and 9.4% of isolates were harbored *ermC*, *ermA*, *ermB* and *ereA*, respectively. Danelli et al.. (2020) reported that the majority of ERY-resistant isolates (82.8%, 77/93) exhibited the iMLSB phenotype, while a small proportion (3.2%, 3/93) displayed cMLSB, and the remaining 14.0% (13/93) fell in the MS category [17].

Consistent with Tabandeh et al.. (2022), the current study found that the cMLSB, iMLSB, and MS phenotypes were present in 61.1%, 22.2%, and 14.8% of isolates, respectively [26].

The prevalence of inducible-resistance genes in the MRSA isolates (*n* = 97) was *ermA* (21.6%), *ermB* (16.5%), *ermC* (44.3%), and *ereA* (9.3%). However, studies by Solgi et al. [28]. , Khodabandeh et al. [4]. , , Gupta et al. [29]. , , Adhikari et al. [30]. , , Ruiz-Ripa et al. [31]. , , and Deotale et al. [32]. , , reported conflicting results regarding the prevalence of these genes.

In this study, inducible-resistant strains were significantly higher in MRSA as well as biofilm producing strains. In total, 50.0% (n; 16/32), 28.1% (n; 9/32) and 21.8% (n; 7/32) of isolates have strong, weak and moderate biofilm, respectively. In this regards the prevalence of biofilm genes was as follows; *icaD* (n; 26/32, 81.3%), *icaA* (n; 22/32, 68.7%), *icaC* (n; 19/32, 59.4%) and *icaB* (n; 14/32, 43.7%). All MRSA strains carried *icaA* gene.

Consistent with the findings of Tabandeh et al.. (2022), the 97 MRSA isolates in our study exhibited the following prevalence of ica genes: *icaA* (84.5%), *icaB* (70.1%), *icaC* (74.2%), and *icaD* (81.4%) [26]. In a similar study conducted by Sedaghat et al., (2018) the prevalence of *icaA* gene was 74.0% (n; 39/53) and 72.0% (n; 18/25) and *icaD* was 81.0% (n; 43/53) and 64.0% (n; 16/64) in MRSA and MSSA, respectively [33].

Contrary to our study, Omidi et al., (2020) showed that 76.0% (n; 111/146) and 87.5% (n; 21/24) of *S. aureus* and MRSA strains were able to strong biofilm production, respectively. 75% (*n* = 18/24) of MRSA isolates tested were found to possess the *icaA* gene, whereas no *icaD* gene was detected [34]. This difference can be due to the presence of genes other than *ica* that play a role in biofilm formation. Biofilm formation by *S. aureus* has been suggested to be primarily driven by the PIA pathway, encoded by the ica operon. However, there is evidence of an ica-independent pathway, linked to the expression of Bap [35]. In addition, it has been observed that methicillin resistance is linked to the inhibition of PIA and biofilm formation dependent on surface proteins.

## Conclusion

Our findings indicate a considerable prevalence of MRSA colonization among HCWs, highlighting a persistent and significant healthcare challenge within our region. Conversely, the management of antibiotic prescriptions to mitigate selective pressures is essential for addressing the emergence of multidrug-resistant (MDR) isolates, including vancomycin-intermediate *Staphylococcus aureus* (VISA) and vancomycin-resistant *Staphylococcus aureus* (VRSA) strains. To enhance the management of *S. aureus* infections, techniques for avoiding biofilm development and dissolving existing biofilms should be investigated.

### Limitations

This study is subject to certain limitations: the primary limitation pertains to the incomplete availability of comprehensive patient history background information. Furthermore, it is imperative to acknowledge that the isolation of MRSA strains was confined to different hospitals, necessitating a cautious approach to the interpretation of the results.

## Data Availability

No datasets were generated or analysed during the current study.

## Abbreviations

SSTIs: skin and soft tissue infections
HCWs: healthcare workers
MRSA: methicillin- resistant Staphylococcus aureus
ERY: erythromycin
CD: Clindamycin
iMLSB: inducible MLSB phenotype
cMLSB: constitutive resistance
MLSB Bap: biofilm-associated proteins
PIA: polysaccharide intercellular adhesive
